# Real-World Experience with Brolucizumab in Wet Age-Related Macular Degeneration: The REBA Study

**DOI:** 10.3390/jcm10132758

**Published:** 2021-06-23

**Authors:** Alper Bilgic, Laurent Kodjikian, Francesc March de Ribot, Vaishali Vasavada, Jesus H. Gonzalez-Cortes, Amro Abukashabah, Aditya Sudhalkar, Thibaud Mathis

**Affiliations:** 1 Alphavision Augenarztpraxis, 27568 Bremerhaven, Germany; drbilgicalper@yahoo.com; 2 Service d’Ophtalmologie, Centre Hospitalier Universitaire de la Croix-Rousse, Hospices Civils de Lyon, Université Claude Bernard Lyon 1, 69004 Lyon, France; laurent.kodjikian@chu-lyon.fr (L.K.); dr.heartaaa@hotmail.com (A.A.); thibaud.mathis@chu-lyon.fr (T.M.); 3 UMR-CNRS 5510, Matéis, Villeurbane, 69004 Lyon, France; 4 Department of Ophthalmology, Universitat Autonoma de Barcelona, 08003 Barcelona, Spain; marchfrancesc@gmail.com; 5 Raghudeep Eye Hospital, Ahmedabad 380054, India; vaishali@raghudeepeyeclinic.com; 6 Department of Ophthalmology, Universitat Autonoma de Ciudad, Mexico City 06720, Mexico; drjesusgzz@gmail.com; 7 Ophthalmology Department, King Abdulaziz University, Rabigh 25732, Saudi Arabia; 8 MS Sudhalkar Medical Research Foundation, Baroda 390001, India

**Keywords:** age-related macular degeneration, anti-vascular endothelial growth factor, brolucizumab, exudation, switch therapy

## Abstract

The aim of the present study was to determine the efficacy and safety of intravitreal brolucizumab therapy for neovascular age-related macular degeneration (AMD) in the real-world setting. The REBA study (real-world experience with brolucizumab in wet AMD) was a retrospective, observational, multicentric study that included 78 consecutive patients (105 eyes), with neovascular AMD, who received brolucizumab therapy. Both treatment-naive and switch-therapy patients were included. Switch therapy was based either on fluid recurrence, fluid recalcitrance, or inability to extend beyond q4/q6. All relevant data were collected. The primary outcome measure was change in best-corrected visual acuity (BCVA) over time. Secondary outcome measures included determination of change in central subfield thickness (CST) and complications. The mean baseline BCVA was 49.4 ± 5.4 letters and 40 ± 3.2 letters, and corresponding mean BCVA gain was +11.9 ± 3.9 letters (*p* = 0.011) and +10.4 ± 4.8 letters (*p* = 0.014) in the treatment-naive and switch-therapy groups, respectively. The change in CST was significantly decreased in the treatment-naive (*p* = 0.021) and the switch-therapy (*p* = 0.013) groups. The mean follow-up was 10.4 months in both groups. One patient in the switch-therapy group developed vascular occlusion and another a macular hole after the fifth brolucizumab injection. Both patients recovered uneventfully. In conclusion, patients showed a very good anatomical and functional response to brolucizumab therapy in the real world, regardless of prior treatment status, until the end of the follow-up period. Two significant untoward events were noted.

## 1. Introduction

Anti-vascular endothelial growth factor (VEGF) agents have been established as the treatment of choice for neovascular age-related macular degeneration (AMD) [1,2]. Monthly injections are rather impractical and negate functional benefits through intense follow-up schedules. There are psychological and physical consequences [3] due to the strict schedules and the potential threat of geographic atrophy (although it rarely, if ever, manifests) [4]. One way to reduce the number of treatments is infrequent injections (e.g., pro re nata injections, or some manner of treat-and-inject regimen) [5,6,7,8,9] without compromise on visual outcomes. Another approach would be to look at more potent formulations that obviate the need for intense therapy.

The HAWK and HARRIER studies established the non-inferiority of the new molecule brolucizumab vis-a-vis aflibercept, with some analyses suggesting a superior anatomic outcome [10]. Nearly 50% of enrolled patients could receive 12-weekly injections, considerably reducing the treatment burden. However, little is known in patients already treated for neovascular AMD who have been switched to brolucizumab therapy. Moreover, concerns about safety with special reference to intraocular inflammation and vasculitis dampened the initial enthusiasm for the drug [11]. As data continues to evolve, the risk of serious adverse events is currently fixed at 4.6% and continues to be updated [12,13].

The current analysis offers a real-world perspective on patients with wet AMD who received brolucizumab either as primary or as switch therapy.

## 2. Materials and Methods

The REBA study (real-world experience with brolucizumab in wet AMD) was an observational, retrospective, multicentric study conducted at Alphavision Augenarztpraxis, Bremerhaven, Germany and the Raghudeep and Sudhalkar group of eye hospitals, Ahmedabad and Baroda, India. A database search was made for patients with macular neovascularization (MNV) who received brolucizumab as intravitreal therapy. This study complied with the tenets of the Declaration of Helsinski and was approved by an international review board (Ethics Committee of the French Society of Ophthalmology, IRB 00008855 Société Française d’Ophtalmologie IRB#1) and by the ethics committee for the Raghudeep Eye Hospital, Ahmedabad, India. Patients provided informed consent for participation in the study.

### 2.1. Eligibility

We looked at the outcomes in treatment-naive patients with neovascular AMD who received intravitreal brolucizumab as well as those who received brolucizumab as switch therapy. Patients who received switch therapy had to have received prior therapy in accordance with the treat-and-extend protocol with either ranibizumab or aflibercept to ensure that they had been treated with current guidelines. Therapy switch was based on previously published recommendations [14]. Patients with polypoidal choroidal vasculopathy (PCV) or retinal angiomatous proliferation (RAP) were excluded.

### 2.2. Definitions

Standard definitions in compliance with the latest consensus guidelines [15] are reiterated here for ease of interpretation:

- Intraretinal fluid (IRF): accumulation of the fluid in retinal thickening and formation of cystoid spaces.
- Subretinal fluid (SRF): leakage in excess of the local capability of removal leading to accumulation of the fluid under the retina.
- Retinal pigment epithelial detachment (PED): a clinically evident separation of the retinal pigment epithelium (RPE) monolayer from the underlying Bruch’s membrane.

Fluid at the macula was identified as intraretinal or subretinal fluid, whereas its disappearance post-injection was considered to be a ‘fluid-free macula or a complete response’ [7]. PED fluid was noted if present, but it was not considered to be an independent treatment criterion as per the HAWK and HARRIER trials. Baseline images were graded independently by two of the investigators (F.M.R. and A.B.) and adjudicated by a senior colleague (L.K.).

### 2.3. Grouping

Treatment-naive patients with macular neovascularization were assessed separately from those who had received brolucizumab as switch therapy. Switch patients did not receive the loading phase of three injections; therapy was based on clinical signs, symptoms, and spectral-domain optical coherence tomography (SD-OCT) scans. Patients in the switch-therapy group were divided into three subgroups according to the reason for the switch:

- Recurrent group (subgroup 1): good effectiveness of the previous therapy (>100 µm reduction in fluid on SD-OCT at day 15 with recurrence at day 30).
- Recalcitrant group (subgroup 2): no reduction (<100 µm reduction in fluid on SD-OCT at days 15 and 30) or worsening disease.
- Inability-to-extend group (subgroup 3): patients for whom the therapy could not be extended after q4/q6 without fluid recurrence.

### 2.4. Acquisition of Data

Data retrieved included patient demographics, the best corrected visual acuity as recorded using the Early Treatment Diabetic Retinopathy Study (ETDRS) chart (also mentioned in the manuscript in Snellen’s notations for ease of interpretation), the best-corrected visual acuity (BCVA), intraocular pressure (IOP), the details of the ocular examination and special investigations conducted, such as fluorescein angiography (FA) and/or indocyanine angiography (ICGA) and central subfield thickness (CST) as determined by SD-OCT, the type of MNV (type 1/type 2/mixed), the size and location thereof, the anti-VEGF agents used, the number of injections administered, the treatment-free interval, and the need for a therapy switch, if any. BCVA, measurement of IOP, slit-lamp examination, fundoscopy, and SD-OCT were documented at each visit.

### 2.5. Follow-Up

**Figure 1:**
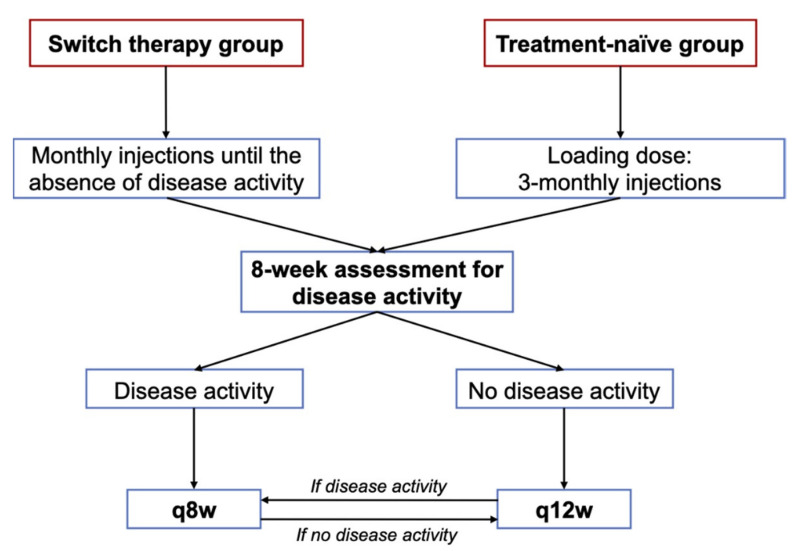
Our in-house protocol for the treatment of wet AMD with intravitreal injections of brolucizumab.

Follow-up was performed according to an in-house protocol for the follow-up of patients under brolucizumab and in accordance with the recommendation for patients under anti-VEGF injections (Stellungn./Empfehlungen DOG). Eyes were examined for detection of ocular-adverse events on days 7, 15, and 30 following the first injection (including OCT scans at day 15) and every month thereafter. After the loading dose, all treatment-naïve patients were assessed at 8 weeks: (i) If no disease activity (i.e., presence of fluid) was observed the patient still had an injection at 12-weeks, and was then reassessed every 12-weeks with another systematic injection (q12w). In cases of disease activity in a follow-up visit, the delay between injections was shortened to 8 weeks. (ii) If fluid was observed, the patient had an injection at 8 weeks and was then reassessed 8-weekly with a systematic injection (q8w). If no fluid was detected at an 8-week follow-up, extension of injections and follow-up could be done at 12 weeks. For the switch-therapy group, patients were followed monthly after the first injections for possible reinjection in case of disease activity. Once the macula was dry on SD-OCT, all switch patients were assessed at 8 weeks and were reinjected following the above recommendations (Figure 1). Retreatment criteria were based on comparisons with the previous month’s examination and retreatment administered if any one criterion of disease activity was considered to be positive [7,10]. Intravitreal injections were performed using a standardized aseptic technique.

Patients had to be followed for a minimum of 9 months to be included in this study. Moreover, for the treatment-naïve group, eyes had to have received at least four intravitreal injections of brolucizumab.

### 2.6. Outcome Measures

The primary outcome measure was to determine the change in BCVA from baseline with treatment. Secondary outcome measures included change in CST in SD-OCT, the mean number of injections required to achieve repeat resolution, and the complications associated therewith. We also attempted to determine which factors independently influence resolution of exudation with a single injection.

### 2.7. Statistical Analysis

The description of categorical variables was based on absolute (size) and relative (percentage) frequencies. Quantitative variables were represented as mean and standard deviation. The comparison of the categorical variables between the groups of different indications was performed using Fisher’s exact test, and when the pairwise comparisons were subsequently performed, the *p*-value was adjusted using the Benjamini–Hochberg method, wherever applicable. When more than two groups were compared, ANOVA was used. A *p*-value < 0.05 was considered to be statistically significant. Quarterly visits were considered to make up for possible lost follow-ups.

## 3. Results

A total of 85 patients (112 eyes) have received intravitreal brolucizumab at our centres thus far, of which 78 patients (105 eyes) have completed at least 9 months of follow-up. The remaining seven patients (seven eyes; all switch-therapy patients) did not complete 9 months of follow-up. These 78 patients form the basis for our analysis (68 patients were of Caucasian ethnicity and included in Germany centers and 10 patients were of south Asian ethnicity and included in Indian centers). Of these 78 patients, 23 patients (25 eyes) were treatment-naïve at baseline whereas the rest received switch therapy. The total number of intravitreal procedures carried out in these patients was 572. Table 1 lists salient characteristics of patients in both groups i.e., treatment-naïve and patients who received switch therapy.

### 3.1. Treatment-Naïve Group

A total of 23 patients (25 eyes) were treated for the first time with brolucizumab (all of Caucasian/European ethnicity). The mean time to presentation after onset of symptoms in the treatment-naïve group was 43.2 ± 12.5 days. Baseline BCVA was 49.4 ± 4.5 letters and the mean retinal thickness was 428.1 ± 73.4 µm. Type 1 MNV was the most frequent neovascular lesion (56.0%). The mean neovascular membrane size was 188.4 ± 44.5 µm.

Overall, 19/25 (76.0%) eyes showed completely resolved exudation at the end of the loading phase and were planned to undergo injections every 12 weeks (q12w), 13/19 (68.4%) eyes were still treated with q12w dosing at the end of follow-up, and 6/13 (46.1%) eyes did not show any recurrence with the q12w regimen (i.e., no disease activity detected at any time). A total of 6/25 patients were injected at 8-weeks (q8w) after the loading phase. For these patients, none of them experienced fluid at the 4-weeks SD-OCT. In this q8w subgroup, 3/6 eyes were finally extended to q12w dosing at the end of follow-up.

The mean final CST decreased significantly to 278.0 ± 47.2 µm (*p* = 0.021) at the final follow-up. The mean follow-up in the treatment-naïve group was 10.4 ± 1.5 months. Patients received a mean of 2.1 ± 0.8 brolucizumab injections after the three loading-dose injections.

### 3.2. Visual Gain

The mean letter gain was 11.9 ± 3.8 letters in this group (*p* = 0.011). A total of 15/25 eyes (60%) gained more than 15 letters from baseline at 8 weeks after the loading phase. Of these eyes, 14/25 maintained the 15-letter gain until the end of the follow-up period. Overall, 16/25 (64.0%) patients retained a BCVA of 20/30 or better at the end of the follow-up period. None of the treatment-naive group lost letters from baseline.

### 3.3. Recurrence

A total of 17/25 (68.0%) eyes demonstrated a recurrence in exudation during the whole follow-up period; 7/17 demonstrated a recurrence when treated at 12 weeks whereas 10/17 eyes demonstrated fluid at 8 weeks. Patients who showed a recurrence tended to have type 1 MNV, higher MNV size (mean 287.4 ± 42.5 µm; *p* = 0.03) and peripapillary MNVs. The presence of IRF was more likely to indicate recurrence (OR-2.31, CI 1.7–4.18; *p* = 0.012).

### 3.4. Switch-Therapy Group

A total of 55 patients (80 eyes; 45 patients were of Caucasian/European, and 10 of Asian ethnicity) received switch therapy from either aflibercept or ranibizumab to brolucizumab. All neovascular AMD patients receiving brolucizumab as switch therapy had been initiated on and received aflibercept injections as primary therapy. According to the cause of the switch, 25 eyes were classified in the subgroup 1 (recurrent), 44 eyes in the subgroup 2 (recalcitrant), and 11 in the subgroup 3 (inability to extend). The mean number of injections prior to switch was 32.4 (±3.4; range 12–96). The minimum number of injections prior to the switch was 12.

Baseline BCVA was 40.0 ± 3.2 letters and the mean retinal thickness was 483.2 ± 59.2 µm. Type 1 MNV was the most frequent neovascular lesion (70.0%). Out of the 80 eyes included in this group, 27 (33.7%) were considered to have no disease activity after the first injection, 38 (47.5%) after the second injection, and 15 (18.8%) after the third injection. None of the patients required a fourth monthly injection before the extension at 8 weeks.

**Figure 2:**
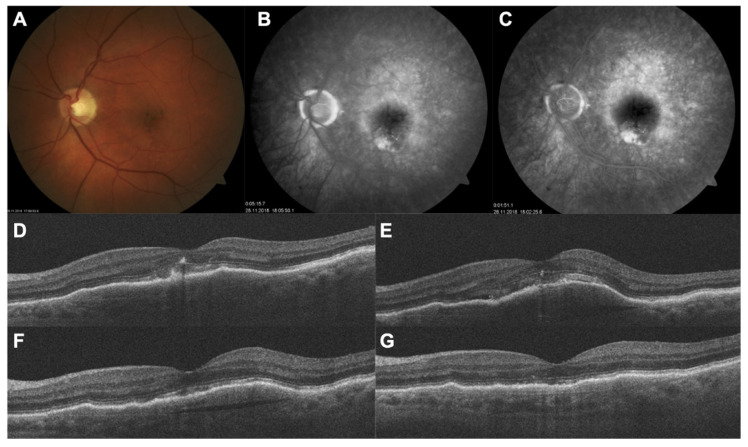
A 74-year-old male with neovascular AMD was switched to brolucizumab after demonstrating worsening of anatomical changes despite six consecutive injections of aflibercept followed by six consecutive injections of ranibizumab. (**A**–**D**) Multimodal imaging showing the baseline situation before any anti-VEGF therapy. (**E**) SD-OCT showing the condition of the retina immediately prior to initiation of brolucizumab therapy. Large pigment epithelium detachment (PED) was present, associated with persistent subretinal fluid (SRF). (**F**) SD-OCT demonstrating significant improvement after the first intravitreal brolucizumab injection and further resolution of PED and SRF. (**G**) SD-OCT after the second injection. The patient has continued to receive therapy in the q12w dosing regimen.

The treatment interval was extended to 12 weeks (q12w) in 25/80 (31.2%) eyes at the end of the follow-up period and 55/80 (68.8%) eyes were maintained at 8 weeks (q8w). The mean CST decreased significantly to 297.5 ± 53.4 µm (*p* = 0.01) at the final follow-up. The mean follow-up in the switch-therapy group was 10.4 ± 2.2 months and patients received a mean of 4.2 ± 2.1 brolucizumab injections during the follow-up period (Figure 2).

### 3.5. Visual Gain

The mean letter gain was 10.4 ± 4.8 letters in this group (*p* = 0.014). A total of 8/80 eyes (10%) gained more than 15 letters from baseline at the end of follow-up. Overall, 9/80 (11.2%) patients retained a BCVA of 20/30 or better at the end of the follow-up period, and 2/80 (2.5%) eyes in the switch-therapy group lost letters from baseline.

According to the cause of switch, the visual gain was 10.9 ± 3.3 letters, 8.6 ± 1.2 letters, and 7.2 ± 2.1 letters for the subgroup 1, 2, and 3, respectively (*p* = 0.051; ANOVA).

### 3.6. Adverse Events

**Figure 3:**
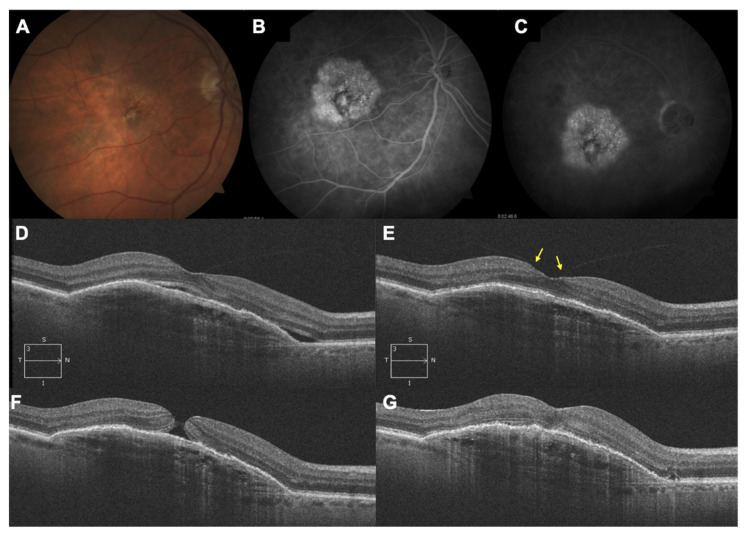
A 78-year-old male patient had been treated for neovascular AMD with aflibercept and ranibizumab and had received 50 injections overall prior to switch to brolucizumab. (**A**–**C**) Multimodal imaging at baseline showing a large subfoveal lesion. (**D**) SD-OCT immediately prior to initiation of brolucizumab therapy. (**E**) SD-OCT 8 weeks after the first brolucizumab injection showing a total resorption of the subretinal fluid but also an evident vitreomacular adherence (arrows). (**F**) SD-OCT 15 days after receiving his 5th brolucizumab injection, showing macular hole. SD-OCT after the macular hole surgery. (**G**) C-late phase FFA.

A total of two patients were noted to have had untoward events after switching to brolucizumab. A 78-year-old male patient (Figure 3) developed a macular hole 15 days after receiving his 5th brolucizumab injection (and 55th injection overall). The patient underwent uneventful macular hole surgery with C_2_F_6_ intraocular gas tamponade and recovered well; his BCVA was 20/80 prior to the development of macular hole. The BCVA had dropped to 20/200 when he was detected to have a macular hole. Post vitrectomy and gas tamponade, his BCVA recovered to 20/100 and has been stable for 4 months (until the last follow-up) after surgery. He has been switched to aflibercept therapy (six injections overall) and has been maintained on q8w dosing.

**Figure 4:**
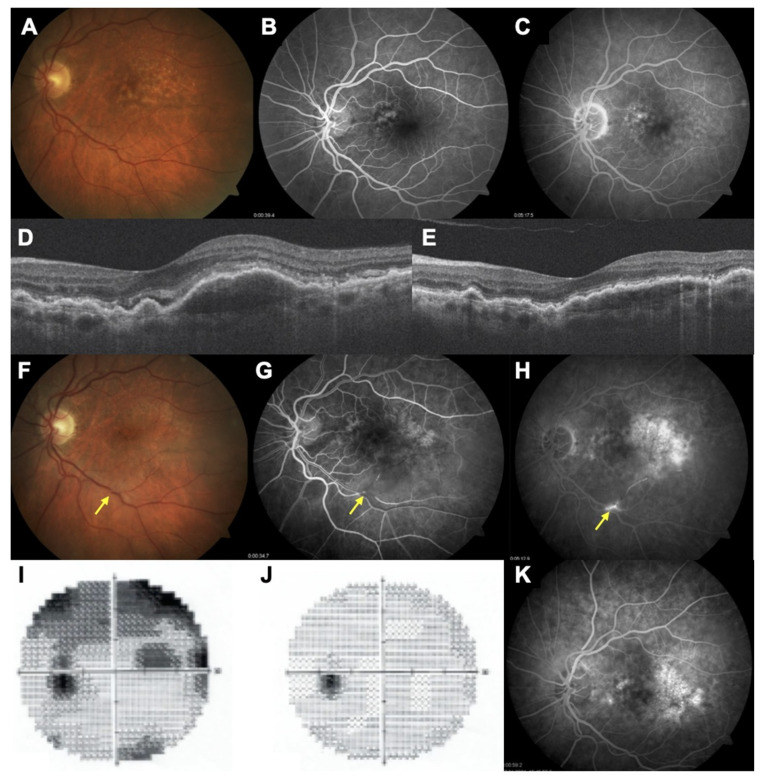
A 71-year-old woman with neovascular AMD had received 70 injections prior to initiation of brolucizumab therapy. (**A**–**C**) Multimodal imaging showing the baseline situation before any anti-VEGF therapy. (**D**) SD-OCT immediately prior to initiation of brolucizumab therapy. (**E**) SD-OCT after the first brolucizumab injection showing total resorption of subretinal fluid and decrease of the pigment epithelium detachment. The patient complained of loss of vision 7 days after the fifth brolucizumab injection. (**F**–**I**) Multimodal imaging showing an retinal arterial occlusion in her left eye (arrow); perimetry analysis demonstrates a corresponding superior scotoma. She had presented within 4 h of onset of symptoms and was immediately referred to the emergency room and received low molecular weight heparin. (**J**,**K**) Post-heparin analysis showing perimetry recovering and arterial reperfusion.

A 71-year-old female developed branch arterial occlusion 7 days after receiving her 5th brolucizumab injection and presented within 4 h of onset of symptoms. Her BCVA prior to this event was 20/60; it dropped to 20/200 secondary to the arterial occlusion. Her perimetry analysis showed corresponding field defects. She was immediately referred to the stroke clinic in Bremerhaven, Germany and received low molecular weight heparin. She showed complete recovery, as confirmed with her restoration of BCVA (20/60) and complete disappearance of all field defects (Figure 4). The indication for switch to brolucizumab was an inability to extend the treatment-free interval beyond 4 weeks. With initiation of brolucizumab therapy itself, the interval could be extended to 8 weeks. She has not had any adverse event since and has been continued on aflibercept therapy on a q4w schedule. She has been asked to maintain a close watch on her vision. We did not note any instance of intraocular inflammation, such as anterior chamber reaction, vitritis, or vasculitis, nor vasculitic occlusion in any of the patients.

27 patients (27 eyes) receiving switch therapy were on IOP-lowering medications at the time of switch to brolucizumab. The switch in therapy did not adversely affect IOP control in any of these 27 patients. None of the treatment-naïve patients were on prior therapy with IOP-lowering medications either at the time of first intravitreal brolucizumab injection or during the follow-up period. There was no rise in IOP noted at any point in time during the course of follow-up in any of the patients included in the study.

The seven eyes excluded from the analysis received a mean of 3.2 (±0.8) injections after switch therapy and did not report any untoward event until the end of follow-up. None of the eyes were switched from brolucizumab to aflibercept (except for the two patients with ocular-adverse events described above).

## 4. Discussion

The current analysis demonstrates very good anatomical, and to a significant extent, visual outcomes following the administration of intravitreal brolucizumab in treatment-naïve or switch-therapy patients in routine practice. Therefore, the primary aim of the study was to describe a real-world cohort of patients with lower baseline BCVA (in comparison to randomized control trials), that showed significant letter gain despite having undergone several treatments in the past. A significant proportion of treatment-naïve and switch patients demonstrated excellent visual and anatomical outcomes that were maintained until the end of the follow-up period. A sizeable proportion of eyes in the treatment-naïve group and the switch-therapy group were maintained on q12w dosing.

The visual gains reported in the present study are higher than what was reported in the HAWK or HARRIER protocols [10]. In our study, treatment-naïve patients had a significantly lower baseline BCVA compared to the HAWK and HARRIER studies, and this could explain the greater visual gain. The ‘ceiling effect’ may thus account for the difference of visual gain when baseline BCVA are not similar. The switch-therapy groups demonstrated very good outcomes, and this could also be explained by the lower baseline BCVA than other series described in the literature. A recent paper on real-world evidence with brolucizumab reported no significant changes in BCVA at final follow-up for a cohort of patient who were mainly switched from another anti-VEGF therapy. However, the baseline BCVA reported in the study was higher than ours at 64.1 letters [11]. Another explanation for the high visual gain in the switch group is the presence of fluid, both IRF and SRF, within the retina at baseline in the majority of patients in this group. Brolucizumab injections efficiently treated the fluid in all cases with one to three injections before the total disappearance of the fluid, making an increase in visual acuity possible. Despite a proactive and intensive protocol with historical anti-VEGF molecules, this subgroup of patients had no or low or an ill-sustained response to the previous therapy. The visual gain provided by brolucizumab injections is probably explained by a greater drying effect on the retina and most importantly, it was also sustained in most patients at 8 weeks. However, it should be noted that the comparison in visual gain, according to the cause of switch, did not show any differences, although there was a trend to better visual gain in eyes with fluid at the time of switch in comparison to patients switched for the inability to extend beyond q6/q8 with previous therapy. This point should be investigated further in a larger cohort of patients.

The smaller size of the brolucizumab molecule means a larger concentration of the drug can be delivered intraocularly. This probably accounts for improved efficacy and durability but may account for a higher incidence of hypersensitivity-like reactions. The incidence of vascular occlusion has been estimated to be 3.4/10000 injections [16]. Biological agents are known to cause hypersensitivity reactions [17]. Overall, enthusiasm for the drug on account of its increased efficacy has been offset somewhat by reports of increased propensity towards inflammatory adverse events [18,19]. Notwithstanding, the drug is a useful alternative to currently available anti-VEGF agents for wet AMD and indeed can eventually be the drug of choice given its potency. As regards complications, both adverse events were noted in patients receiving switch therapy and occurred after the loading phase. Treatment-naïve patients did not demonstrate adverse events until the last follow-up. One patient developed vascular occlusion after the fifth brolucizumab injection and this was reversed through timely intervention. There were no residual deficits noted, either in terms of visual acuity or perimetric changes. The time to development of this adverse event is contrary to past reports on vasculitis associated with brolucizumab that suggest that such adverse events are noted later (day 30) in the course of the follow-up. Additionally, this occurred after the fifth injection of brolucizumab and the 75th injection overall (including past therapy). Whereas a recently published study comprising 172 eyes receiving brolucizumab and 6 months of follow-up reported 14 instances of intraocular inflammation, only one serious adverse event (vasculitic occlusion) was reported [12]. In the remaining 13 eyes in the study, the vast majority of eyes showed spontaneous resolution whereas some eyes needed intervention in the form of topical or subtenon steroid therapy. The incidence of serious adverse events matches our analysis. Moreover, we report for the first time, to the best of our knowledge, macular hole formation after brolucizumab therapy. The patient responded well to surgery. Macular hole formation has been noted earlier with ranibizumab and bevacizumab [20,21]. A combination of alterations in retinal tractional forces and contracture of the choroidal neovascular membrane consequent to VEGF withdrawal along with altered vitreous dynamics consequent to repeat violations of the milieu interior of the vitreous cavity leads to macular hole formation. Whether the higher molecular concentration of brolucizumab plays any role in its incidence merits further investigation.

The retrospective nature of our study is an obvious limitation in that follow-ups are liable to be missed. This is evolving data as it is a new drug. We attempted to analyze response by ethnicity but the numbers are too small (especially for south Asian ethnicity) to permit meaningful analysis. Notwithstanding, this current analysis is, in our opinion, an accurate portrayal of the current status of intravitreal brolucizumab therapy for neovascular AMD and the challenges associated therewith in the real world as opposed to the setting of a randomized trial. Brolucizumab was effective both in treatment-naïve patients and those receiving switch therapy in terms of visual gain and anatomical resolution of MNV associated exudation and in extension of the treatment interval. There was significant improvement in vision from baseline in both groups, thereby demonstrating its efficacy even in patients who had received multiple injections earlier and in patients with low baseline BCVA. Our findings correspond to early reports on the use of brolucizumab [22] but additionally, we also report on patients receiving switch therapy. The current general consensus the world over is one of reduction in the treatment burden. The FLUID study, for example also looks at how much fluid can be tolerated without much compromise of final vision [23] whereas our recent publication looks at a particular subset of patients who do well with one injection over a considerable period of time [24]. This is also important in reducing treatment costs. Finally, our short case series on the efficacy of brolucizumab in patients with MNV and RPE rip reaffirms to some extent the visual gains reported in the present analysis, albeit a very small number of patients [25].

To conclude, intravitreal brolucizumab therapy is effective in both treatment-naïve patients and patients who receive the drug as switch therapy. There is a small but definite risk associated with the drug (1.9% overall in our series; 0.95% as far as vascular occlusion is concerned). Notwithstanding the small risk of untoward events, intravitreal brolucizumab therapy is a useful addition to the retinal surgeon’s armamentarium and should establish itself as an effective form of primary therapy that can do well with infrequent administration. A high index of suspicion maintained both by the physician and the patient can help avert disastrous complications.

## Figures and Tables

**Table 1:** Characteristics of patients who received brolucizumab therapy.

Characteristic	Treatment-Naïve (N = 25)	Switch Therapy (N = 80)
Mean age, years (SD)	69.2 (4.4)	74.3 (5.8)
Male:female	9:14	26:29
Mean follow-up, months (SD)	10.4 (1.5)	10.4 (2.2) *
Mean baseline BCVA, letters (SD)	49.4 (4.5)	40.0 (3.2)
Mean baseline CST, µm (SD)	428.1 (7.4)	483.2 (59.2)
MNV subtype, *n*:		
Type 1	14	56
Type 2	7	14
Mixed	4	10
MNV localization, *n*:		
Subfoveal	15	56
Juxtafoveal	4	12
Extrafoveal	1	2
Interpapillomacular	5	10
Fluid localization, ** *n*:		
IRF	23	57
SRF	12	31
PED	10	24
None	2	0

BCVA, best corrected visual acuity; IRF, intraretinal; MNV, choroidal neovascularization; PED, pigment epithelium detachment; SRF, subretinal fluid. * After initiation of brolucizumab therapy; ** patients could have fluid in more than one compartment.

## Data Availability

All data are available upon request to the corresponding author.

## References

[1] Rosenfeld P.J., Brown D.M., Heier J.S., Boyer D.S., Kaiser P., Chung C.Y., Kim R.Y. (2006). Ranibizumab for Neovascular Age-Related Macular Degeneration. N. Engl. J. Med..

[2] Brown D.M., Michels M., Kaiser P., Heier J.S., Sy J.P., Ianchulev T. (2009). Ranibizumab versus Verteporfin Photodynamic Therapy for Neovascular Age-Related Macular Degeneration: Two-Year Results of the ANCHOR Study. Ophthalmology.

[3] Boyer D.S., Heier J.S., Brown D.M., Francom S.F., Ianchulev T., Rubio R.G. (2009). A Phase IIIb Study to Evaluate the Safety of Ranibizumab in Subjects with Neovascular Age-related Macular Degeneration. Ophthalmology.

[4] Bhisitkul R.B., Mendes T.S., Rofagha S., Enanoria W., Boyer D.S., Sadda S.R., Zhang K. (2015). Macular Atrophy Progression and 7-Year Vision Outcomes in Subjects from the ANCHOR, MARINA, and HORIZON Studies: The SEVEN-UP Study. Am. J. Ophthalmol..

[5] Singer M.A., Awh C.C., Sadda S., Freeman W.R., Antoszyk A.N., Wong P., Tuomi L. (2012). HORIZON: An Open-Label Extension Trial of Ranibizumab for Choroidal Neovascularization Secondary to Age-Related Macular Degeneration. Ophthalmology.

[6] Gupta B., Adewoyin T., Patel S.K., Sivaprasad S. (2011). Comparison of two intravitreal ranibizumab treatment schedules for neovascular age-related macular degeneration. Br. J. Ophthalmol..

[7] Lalwani G.A., Rosenfeld P.J., Fung A., Dubovy S.R., Michels S., Feuer W., Davis J.L., Flynn H.W.J., Esquiabro M. (2009). A variable-dosing regimen with intravitreal ranibizumab for neovascular age-related macular degeneration: Year 2 of the PrONTO Study. Am. J. Ophthalmol..

[8] Fung A.E., Lalwani G.A., Rosenfeld P.J., Dubovy S.R., Michels S., Feuer W.J., Puliafito C.A., Davis J.L., Flynn H.W., Esquiabro M. (2007). An Optical Coherence Tomography-Guided, Variable Dosing Regimen with Intravitreal Ranibizumab (Lucentis) for Neovascular Age-related Macular Degeneration. Am. J. Ophthalmol..

[9] Schmidt-Erfurth U., Eldem B., Guymer R., Korobelnik J.F., Schlingemann R.O., Axer-Siegel R., Wiedemann P., Simader C., Gekkieva M., Weichselberger A. (2011). Efficacy and safety of monthly versus quarterly ranibizumab treatment in neovascular age-related macular degeneration: The EXCITE study. Ophthalmology.

[10] Dugel P.U., Singh R.P., Koh A., Ogura Y., Weissgerber G., Gedif K., Jaffe G.J., Tadayoni R., Schmidt-Erfurth U., Holz F.G. (2020). HAWK and HARRIER: Ninety-Six-Week Outcomes from the Phase 3 Trials of Brolucizumab for Neovascular Age-Related Macular Degeneration. Ophthalmology.

[11] Enríquez A.B., Baumal C.R., Crane A.M., Witkin A.J., Lally D.R., Liang M.C., Enríquez J.R., Eichenbaum D.A. (2021). Early Experience with Brolucizumab Treatment of Neovascular Age-Related Macular Degeneration. JAMA Ophthalmol..

[12] Baumal C.R., Bodaghi B., Singer M., Tanzer D.J., Seres A., Joshi M.R., Feltgen N., Gale R. (2020). Expert Opinion on Management of Intraocular Inflammation, Retinal Vasculitis, and/or Vascular Occlusion after Brolucizumab Treatment. Ophthalmol. Retina.

[13] Monés J., Srivastava S.K., Jaffe G.J., Tadayoni R., Albini T.A., Kaiser P.K., Holz F.G., Korobelnik J., Kim I.K., Pruente C. (2020). Risk of Inflammation, Retinal Vasculitis, and Retinal Occlusion-Related Events with Brolucizumab: Post Hoc Review of HAWK and HARRIER. Ophthalmology.

[14] Spaide R.F., Jaffe G.J., Sarraf D., Freund K.B., Sadda S.R., Staurenghi G., Waheed N.K., Chakravarthy U., Rosenfeld P.J., Holz F.G. (2020). Consensus Nomenclature for Reporting Neovascular Age-Related Macular Degeneration Data: Consensus on Neovascular Age-Related Macular Degeneration Nomenclature Study Group. Ophthalmology.

[15] Ehlken C., Jungmann S., Böhringer D., Agostini H.T., Junker B., Pielen A. (2014). Switch of anti-VEGF agents is an option for non-responders in the treatment of AMD. Eye.

[16] Novartis Is Confident that Beovu Continues to Represent an Important Treatment Option for Patients with Wet AMD, with an Overall Favorable Benefit/Risk Profile When Used on an 8- to 12-week Interval Following 3 Monthly Loading Doses. https://www.brolucizumab.info.

[17] Puxeddu I., Caltran E., Rocchi V., Del Corso I., Tavoni A., Migliorini P. (2016). Hypersensitivity reactions during treatment with biological agents. Clin. Exp. Rheumatol..

[18] Baumal C.R., Spaide R.F., Vajzovic L., Freund K.B., Walter S.D., John V., Rich R., Chaudhry N., Lakhanpal R.R., Oellers P.R. (2020). Retinal Vasculitis and Intraocular Inflammation after Intravitreal Injection of Brolucizumab. Ophthalmol..

[19] Kabanarou S.A., Xirou T., Mangouritsas G., Garnavou-Xirou C., Boutouri E., Gkizis I., Chatziralli I. (2017). Full-thickness macular hole formation following anti-VEGF injections for neovascular age-related macular degeneration. Clin. Interv. Aging.

[20] Miura M., Iwasaki T., Goto H. (2011). Macular hole formation after intravitreal bevacizumab administration in a patient with myopic choroidal neovascularization. Retin. Cases Brief Rep..

[21] Grigoropoulos V., Emfietzoglou J., Nikolaidis P., Theodossiadis G., Theodossiadis P. (2010). Full-Thickness Macular Hole after Intravitreal Injection of Ranibizumab in a Patient with Retinal Pigment Epithelium Detachment and Tear. Eur. J. Ophthalmol..

[22] Sharma A., Kumar N., Parachuri N., Sadda S.R., Corradetti G., Heier J., Chin A.T., Boyer D., Dayani P., Arepalli S. (2021). Brolucizumab—early real-world experience: BREW study. Eye.

[23] Arnold J.J., Markey C.M., Kurstjens N.P., Guymer R.H. (2016). The role of sub-retinal fluid in determining treatment outcomes in patients with neovascular age-related macular degeneration--a phase IV randomised clinical trial with ranibizumab: The FLUID study. BMC Ophthalmol..

[24] Bilgic A., Kodjikian L., Mathis T., Sudhalkar A.A., Vasavada S.A., Bhojwani D.M. (2021). Single Injection Response to Anti-Vascular Endothelial Growth Factor Agents in Patients with wet Age related Macular Degeneration: Incidence and Characteristics. Retina.

[25] Bilgic A., Kodjikian L., Vasavada S., Jha S., Srivastava S., Sudhalkar A., Mathis T. (2021). Brolucizumab for Choroidal Neovascular Membrane with Pigment Epithelial Tear and Subretinal Fluid. J. Clin. Med..

